# Association between Skin-to-Skin Contact Duration after Caesarean Section and Breastfeeding Outcomes

**DOI:** 10.3390/children9111742

**Published:** 2022-11-12

**Authors:** Juan Juan, Xiaosong Zhang, Xueyin Wang, Jun Liu, Yinli Cao, Ling Tan, Yan Gao, Yinping Qiu, Huixia Yang

**Affiliations:** 1 Department of Obstetrics and Gynecology, Peking University First Hospital, Beijing 100034, China; 2 Department of Obstetrics and Gynecology, Northwest Women’s and Children’s Hospital, Xi’an 710003,China; 3 Department of Obstetrics and Gynecology, Sichuan Provincial Hospital for Women and Children, Chengdu 610045, China; 4 Department of Neonatology, General Hospital of Ningxia Medical University, Yinchuan 750003, China

**Keywords:** skin-to-skin contact, caesarean section, early initiation of breastfeeding, exclusive breastfeeding

## Abstract

We aimed to explore the association between skin-to-skin contact (SSC) duration after caesarean sections (CSs) and breastfeeding outcomes. A prospective study was conducted in four hospitals in China during January and August 2021. A total of 679 participants with singleton pregnancy who delivered by elective CS after 37 gestational weeks using epidural or spinal anesthesia were included. Logistic regression was applied to assess the association between SSC duration and early initiation of breastfeeding (EIBF), as well as the promoting factors for exclusive breastfeeding (EBF) at hospital discharge. Immediate SSC after CSs was strongly associated with higher rates of EIBF (*p* < 0.001) and EBF at hospital discharge (*p* = 0.002). The EIBF rates increased with longer duration of SSC, with the at least 90 min SSC group having the highest EIBF rate (74.5%). Skin-to-skin contact durations of at least 90 min, 60–89 min and 30–59 min were significantly associated with 8.53 times (OR = 8.53, 95%CI: 4.94–14.72, *P_adj_* < 0.001), 8.04 times (95%CI: 4.68–13.80, *P_adj_* < 0.001) and 6.28 times (95%CI: 3.75–10.51, *P_adj_* < 0.001), respectively, higher EIBF rates compared to those without immediate SSC. After multiple-testing correction, the rates of EBF at hospital discharge were found to be independent of the duration of SSC (*P_adj_* = 0.12). Early initiation of breastfeeding was not a significant predictor of EBF. Our results suggested that SSC is important for EIBF in Chinese baby-friendly hospitals. Skin-to-skin contact should be practiced after CS to promote breastfeeding and providing SSC with longer duration is encouraged to obtain the full benefit; if it is not feasible, a minimum of 30 min SSC could achieve improved EIBF and EBF at discharge.

## 1. Introduction

Breastfeeding is critical to promote maternal and child health. The significant advantages of breastfeeding for newborns’ survival, growth and development have been widely recognized [1]. It has been reported that breastfeeding can protect against child infections and malocclusion, increase intelligence and reduce the risks of becoming overweight and of diabetes. For nursing women, breastfeeding was found to provide protection against breast cancer, ovarian cancer and type 2 diabetes mellitus [1]. The optimal route for breastfeeding recommended by World Health Organization (WHO) and the United Nations Children’s Fund (UNICEF) includes early initiation of breastfeeding (EIBF), exclusive breastfeeding (EBF) for up to 6 months and continuation of breastfeeding for up to 2 years or beyond [2,3]. Optimal breastfeeding practices are associated with reduced newborn and child morbidity and mortality and have potential long-term benefits for both mother and child [4,5,6]. Despite strong evidence for the nutritional and immunological benefits of breastfeeding in promoting maternal and child health, only 48% of newborns globally and 29% in China initiate breastfeeding within the first hour after birth [7]. The rate of EBF was only 37% in low-income and middle-income counties [1]. Improving EBF is a priority worldwide, and all nations are on course to achieve the WHO global nutrition goal of increasing the EBF rate to at least 50% by 2025 [8].

Mother and newborn skin-to-skin contact (SSC) after birth is an effective way to improve breastfeeding initiation and duration [9,10,11,12]. It is recommended that SSC should be facilitated immediately after birth, as this is the time when the newborn is most likely to follow his/her natural instincts to find and attach to the breast and then breastfeed. Immediate SSC is a beneficial practice and promotes breastfeeding outcomes, and it is commonly performed after normal vaginal delivery. In contrast to the well-established evidence for vaginal delivery, high-quality clinical evidence for SSC after caesarean sections (CSs) is limited. Despite the lack of evidence, the global rate of CSs has been increasing steadily over the past few decades, with China reporting a CS rate of 34.9% in 2014 according to a descriptive study conducted in 2865 counties in 31 provinces in mainland China [13]. Caesarean sections are widely believed to reduce the initiation of breastfeeding, increase the time before the first breastfeed, delay the onset of lactation and reduce the rate of EBF, increasing the likelihood of supplementation [14,15,16]. It is important to find ways to improve breastfeeding rates after CSs. The WHO and UNICEF Baby-Friendly Hospital Initiative Implementation Guidance recommend that immediate and uninterrupted SSC is feasible after CSs with epidural anesthesia [17] because most of the mothers and their newborns are healthy and remain alert and responsive under spinal or epidural anesthesia. However, studies on whether SSC is also effective in improving breastfeeding outcomes after CSs and how to effectively provide SSC after CSs to improve breastfeeding are limited. Therefore, we aimed to explore the association between SSC after CSs and breastfeeding outcomes, as well as the optimal duration of SSC after CSs, to provide a scientific basis for implementing SSC after CSs in China

## 2. Methods

### 2.1. Study Participants

A prospective study was conducted in four tertiary hospitals from Beijing, Shaanxi, Sichuan and Ningxia provinces in China during January and August 2021. All four participating hospitals are baby-friendly hospitals and support breastfeeding according to the Ten Steps to Successful Breastfeeding described by the Baby-Friendly Hospital Initiative (BFHI) launched by the UNICEF and WHO. Participants with singleton pregnancy who delivered by elective CS after 37 gestational weeks using epidural or spinal anesthesia were included in this study. Pregnant women who had serious pregnancy complications, such as placenta accrete spectrum or eclampsia; whose newborns suffered from neonatal asphyxia, birth defects or other serious complications; or whose status for SSC and breastfeeding was unknown were excluded from this study. Apart from immediate SSC after CS, all infants received routine care after birth, including immediate drying, thermal care, breastfeeding support, newborn eye care, vitamin K1 (the vitamin K was injected intramuscularly to the infants and the general dosage was 1 mg), immunizations (hepatitis B and BCG vaccinations), weighing, Apgar score and umbilical cord pH evaluation, monitoring of vital signs and temperature and meticulous physical and neurodevelopmental examinations by midwife and neonatologist. Information regarding the maternal and neonatal characteristics of participants, SSC duration, time to exhibition of feeding cues and initiation of breastfeeding, EBF at hospital discharge, etc., as well as the safety of the infant, were collected and recorded continuously during elective CSs by trained staff in the four participating hospitals. 

### 2.2. Outcome Definitions

Skin-to-skin contact was defined as the placement of a dried, naked infant, with the head covered by a dry cap to prevent heat loss, directly on the bare chest of the mother immediately after a CS, without separation and with the exposed back of the infant covered by warm blankets or a towel [6]. Duration of SSC was defined as the period of direct SSC contact between the bare skin of the mother and newborn. Reasons relating to anesthesia, medical reasons and personal reasons, such as severe pain, severe bleeding, etc., might have contributed to the duration of SSC for the participants, and the participants were categorized into five groups according to the actual duration of SSC: no SSC, less than 30 min, 30–59 min, 60–89 min and at least 90 min. Early initiation of breastfeeding was defined as the initiation of the first breastfeed within the first hour of birth. Exclusive breastfeeding at hospital discharge was defined as feeding of only breastmilk without other food or fluids from birth to hospital discharge. Mixed feeding was defined as feeding of breastmilk together with other liquids and/or foods, such as water, formula, or any type of solid food. Formula feeding was defined as feeding of formula without breastmilk. Time to exhibition of feeding cues was defined as the time between birth and exhibition of any of the feeding cues, including rooting toward the mother’s chest, turning of the head, opening and closing mouth, licking the lips, moving hand to mouth, sucking fingers or hands, etc. Time to initiation of breastfeeding was defined as the time between birth and the initiation of the first breastfeeding. 

### 2.3. Statistical Analysis

Characteristics of the participants are presented as means ± standard deviation (SD) for normally distributed continuous variables or medians and the interquartile range (IQR) for skewed distributed continuous variables. Categorical variables were summarized as numbers (percentages). Analysis of variance (ANOVA), the Kruskal–Wallis test or the χ^2^ test were used to compare the differences among different SSC duration groups for normally distributed continuous variables, skewed distributed continuous variables and categorical variables, respectively. Multiple-testing corrections were performed using Bonferroni corrections to adjust probability (*p*) values for breastfeeding outcomes. Univariate and multivariate logistic regression models were performed to assess the association between SSC duration and EIBF and the promoting factors for EBF at hospital discharge. We adjusted for various potential confounding factors, including maternal age, education, delivery gestational week, and parity. Considering potential clustering by study site, we fitted a mixed model, assuming a customized baseline risk in each hospital. All statistical analysis was conducted using SPSS 20.0 statistical software (SPSS Inc., Chicago, Illinois). A two-sided *p* < 0.05 indicated significance.

The study was approved by the Institutional Review Board of Peking University First Hospital, Beijing, China. The study was conducted in accordance with the Declaration of Helsinki. Written informed consent was signed by every participant before they participated in the study.

## 3. Results

A total of 679 participants from four hospitals were included in this study; among them, 136 eligible participants without SSC after CS (20%, 136/679) were categorized as controls and received routine care. In total, 80.0% (543/679) of participants performed immediate SSC after the CS, with 21.4% (145/679) remaining in SSC for at least 90 min after birth. Overall, 58.0% (394/679) of newborns initiated breastfeeding early and the EBF rate at hospital discharge was 62.9% (427/679). The maternal and neonatal characteristics of participants according to SSC duration are shown in Table 1. The mean age of the participants was 32.06 (range 19–44) years and the median delivery gestational week was 39.14 (IQR 37.87–39.43) weeks. More than half (62.7%) of the participants had an undergraduate degree; the rest (16.4% and 20.9%) had a graduate degree or above and a high school degree or less, respectively. The mean birthweight was 3358.75 ± 396.15 g and almost half of the neonates were female. There were no statistically significant differences in maternal characteristics, including age, delivery gestational week, gravidity, parity, education level, height, and weight before delivery, or neonatal characteristics, including birth weight, gender and birth length, among participants with different SSC durations (*p* > 0.05).

### 3.1. Association between SSC after CSs and Breastfeeding Outcomes

Skin-to-skin contact after CSs was strongly associated with higher rates of EIBF (*p* < 0.001) and EBF at hospital discharge (*p* = 0.002). Longer SSC duration after CSs led to an increased rate of EIBF, with the at least 90 min SSC group having the highest rates of EIBF (74.5%), compared to those without SSC (27.2%) (P*adj* < 0.001). Participants who received SSC longer demonstrated earlier median times to exhibition feeding cues and initiation of breastfeeding (P*adj* < 0.001). The rates of EBF at hospital discharge among the less than 30 min SSC group, 30–59 min SSC group, 60–89 min SSC group and at least 90 min SSC group were 61.9%, 66.1%, 64.4% and 69.0%, respectively, and they were higher than 51.5% in the group without SSC (*p* = 0.03). However, after correction for multiple testing, the rates of EBF at hospital discharge were independent of the duration of SSC (P*adj* = 0.12) (Table 2).

### 3.2. Skin-to-Skin Contact Duration after CSs and Early Initiation of Breastfeeding

**Figure 1:**
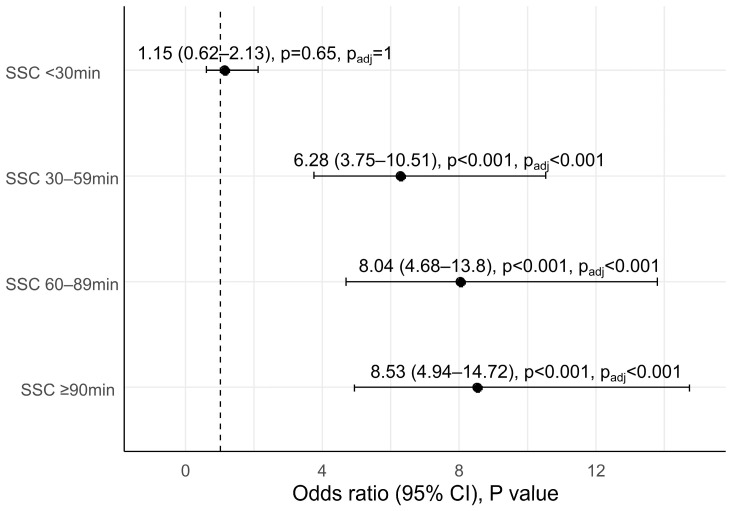
Association between SSC duration after caesarean section and early initiation of breastfeeding.

Longer SSC duration after CSs led to an increased rate of EIBF. After adjustment for potential confounding factors, SSC durations of at least 90 min, 60–89 min and 30–59 min were significantly associated with 8.53 times (95%CI: 4.94–14.72, P_adj_ < 0.001), 8.04 times (95%CI: 4.68–13.80, P_adj_ < 0.001) and 6.28 times (95%CI: 3.75–10.51, P_adj_ < 0.001), respectively, higher EIBF rates compared to those without immediate SSC (Figure 1).

### 3.3. Promoting Factors for Exclusive Breastfeeding at Hospital Discharge

The effects of promoting factors on EBF at hospital discharge are shown in Table 3. Significant associations were found between EBF at hospital discharge and duration of SSC after CS, multiparity, macrosomia and maternal weight before delivery (*p* < 0.05). Longer SSC duration and multiparity were associated with an increased rate of EBF at hospital discharge. Maternal weight before delivery and macrosomia were negatively associated with rate of EBF at hospital discharge. When adjusted for potential confounding factors, except for multiparity (*p* = 0.10), SSC durations of 30–59 min (OR = 1.86, 95% CI: 1.15–3.01, *p* = 0.01), 60–89 min (OR = 1.79, 95% CI: 1.09–2.91, *p* = 0.02), and at least 90 min (OR = 2.16, 95% CI: 1.31–3.56, *p* = 0.002); macrosomia (OR = 0.53, 95% CI: 0.31–0.92, *p* = 0.03); and maternal weight before delivery (OR = 0.96, 95% CI: 0.94–0.97, *p* < 0.001) remained significantly associated with EBF at hospital discharge. Maternal age, time to exhibition of feeding cues and time to initiation of breastfeeding were not significantly associated with EBF at hospital discharge (*p* > 0.05).

## 4. Discussion

In this study, we demonstrated that longer SSC duration after CSs led to an increased rate of EIBF, with the at least 90 min SSC group having the highest rates of EIBF of 74.5% and the no-SSC group having the lowest rates of EIBF (27.2%) (*Padj* < 0.001). Longer SSC duration after CSs led to an increased rate of EIBF. Exclusive breastfeeding rates at hospital discharge ranged from 51.5% for newborns without SSC to 69.0% for newborns with at least 90 min SSC. After multiple-testing correction, the rates of EBF at hospital discharge were independent of the duration of SSC (*P_adj_* = 0.12). Longer SSC duration was associated with earlier median time to exhibition of feeding cues and initiation of breastfeeding (*Padj* < 0.001). 

Breastfeeding provides an ideal source of nutrition for newborns and is widely recognized as one of the most effective measures to ensure newborn survival, growth, development and lifelong health. However, breastfeeding has improved only moderately over time, with just 44% of newborns put to the breast within the first hour after birth globally and disparity in rates across counties [18]. More efforts are needed to achieve the global target of improving rates of EIBF to 70% [18]. Skin-to-skin contact is well-known to enhance breastfeeding outcomes. Moore et al. have shown that SSC improved breastfeeding outcomes following normal vaginal birth in a systematic review [9]. A study of SSC following vaginal delivery conducted in 46 hospitals in 18 counties in four western provinces of China showed that the EBF rate at hospital discharge increased from 43% to 73% [19]. 

Although SSC is a beneficial practice and promotes breastfeeding outcomes [9,10,11,12], most studies have primarily focused on women following vaginal births [9]. There are a limited number of studies reviewing SSC after CS. It is important to determine whether this is also true following a CS, as the rate of CSs has been gradually increasing throughout the world and in China [13]. In addition, CSs are known to limit breastfeeding and impose difficulties that lead to breastfeeding cessation [14,15,16,20]. Women who delivered by CS experienced a significant delay in breastfeeding initiation compared to women who give birth vaginally [21]. A study conducted by Karim et al. of 3162 mothers showed that 51% initiated breastfeeding within the first hour of delivery and women who had CSs were less likely to initiate breastfeeding early after birth than those who underwent vaginal deliveries (OR = 0.32, 95% CI:0.23–0.43, *p* < 0.001) [22]. Another study conducted by Singh et al. showed that newborns delivered by CS had 67% lower odds of EIBF than those who underwent vaginal delivery (OR = 0.33, 95% CI:0.26–0.43) [23]. A systematic review and meta-analysis of 554,568 participants from 33 countries showed that CSs were associated with a significant reduction in early breastfeeding compared to vaginal delivery [16]. A previous study has shown that SSC can be implemented successfully after CSs and is safe for both mothers and newborns [9]. In our study, we found that newborns delivered by CS who received SSC of at least 60 min (74.0%) could achieve the WHO global target of 70%, which was about 2.7 times higher than those who did not receive SSC (27.2%). Likewise, they were more likely to be exclusively breastfed at hospital discharge than those who did not receive SSC, which indicated that SSC could be provided safely and immediately after CSs, and newborns delivered by CS who received SSC were more likely to be breastfed, including initiation within the first hour of birth, and to be exclusively breastfed at hospital discharge. The improvements in breastfeeding were most significant when SSC was maintained for longer times. Therefore, promoting SSC immediately after CSs is an effective way to encourage EIBF and EBF; thus, immediate SSC can be offered to all women regardless of the mode of delivery.

A cross-sectional observational study of 150 national, provincial and district hospitals in eight countries in East Asia and the Pacific reported that the duration of SSC and EIBF had a strong dose–response relationship. SSC durations of at least 90 min, 60–89 min, 30–59 min, 10–29 min and less than 10 min were associated with 368.81 (95% CI: 88.76–1532.38, *p* < 0.001), 155.89 (95% CI:36.65–663.13, *p* < 0.001), 88.03 (95% CI:21.19–365.78, *p* < 0.001), 30.62 (95% CI:7.31–128.22, *p* < 0.001) and 6.06 (95% CI:1.34–27.29, *p* < 0.001) times higher early breastfeeding rates, respectively [24]. The study provided some evidence about the benefits of immediate and uninterrupted SSC for breastfeeding outcomes regardless of delivery mode. However, in that study, most mothers underwent vaginal delivery (82.6%). Only a small proportion of participants performed SSC after CS. Therefore, whether the duration of SSC after CSs also shows a dose–response relationship with the rate of EIBF is still unknown. In addition, the optimal timing and duration of SSC after CSs has not been established yet. Our study found a similar dose–response relationship trend between the duration of immediate SSC after CS and EIBF and suggested that the optimal duration of SSC after CS should be as long as possible to maximize the likelihood of EIBF. However, achieving longer durations of SSC remains challenging, especially after CSs. In conditions where SSC for longer times is not feasible, a minimum of 30 min SSC should be achieved to improve EIBF and EBF at discharge.

Our study also found that SSC after CS was associated with earlier median time to exhibition of feeding cues and initiation of breastfeeding. The initial moments after birth are a sensitive and critical period, as this is the ideal time to start facilitating newborns’ nutritional behavior and establish effective breastfeeding, such as breast crawling and sucking. Immediate and uninterrupted SSC makes it easier for newborns to crawl towards the mother’s nipples, and the newborns’ feeding behaviors strongly respond to tactile, warmth and olfaction stimulation; thus, newborns might start breastfeeding by utilizing their innate behavior, which effectively contributes to initiate breastfeeding actively and successfully [9]. What’s more, newborns secrete catecholamine during the first hour of adaptation in extrauterine life. The high levels of catecholamine immediately after birth make olfactory bulbs in newborns’ noses extremely sensitive to odor cues [10]. In addition, SSC immediately after birth helps transition newborns to the post-uterine environment and increases the likelihood of breastfeeding initiation [25,26]. As is well-known, birth by CS decreases the production of oxytocin and prolactin, the hormones that aid in initiating milk production and which are essential for breastfeeding. Immediate SSC and newborn sucking stimulate and enhance the production of these hormones that assist with breastfeeding [27,28]. Furthermore, newborns born by CS do not acquire maternal vaginal microbes, and SSC after CSs helps microbial colonization of the newborn with maternal skin microbiota [29]. The importance of early SSC also relates to the colostrum, which contains bioactive immune factors that protect newborns against a wide variety of infectious and allergic diseases. Hence, it is recommended that SSC should be facilitated immediately after CSs so as to help the fully active and responsive newborns follow their natural instinct to find and attach to the breast and then initiate breastfeeding within the first hour of birth.

Understanding the factors and determinants of EBF is essential for targeted and effective breastfeeding promotion programs. According to our study, SSC durations of 30–59 min, 60–89 min and at least 90 min were significantly associated with EBF at hospital discharge, which is consistent with the findings for a previous retrospective cohort of healthy term and singleton infants showing that the odds of EBF at hospital discharge were 3.81 (95%CI:3.64–3.99) times greater among those who had SSC than those who did not [30]. Other factors, such as maternal weight before delivery and macrosomia delivery, were negatively associated with the rate of EBF at hospital discharge. As is well-known, maternal overweight status, excessive gestational weight gain, gestational diabetes mellitus and fetal macrosomia are associated with a higher risk for metabolic syndrome in offspring. In this context, breastfeeding could protect the offspring against metabolic syndrome and reduce the risk of becoming overweight and of diabetes [1]. Therefore, our results indicate that breastfeeding interventions should pay particular attention to women with higher weight before delivery and those who deliver macrosomic babies. In addition, pregnant women should be encouraged to maintain optimal weight gain during pregnancy.

This was a relatively large prospective study conducted in four baby-friendly hospitals to demonstrate the association between SSC duration after CS and breastfeeding outcomes. However, some limitations should also be taken into consideration. First, our study was limited to the period before hospital discharge and we did not conduct long-term follow-up after hospital discharge on breastfeeding outcomes up to six months. Therefore, longer follow-up studies are needed to determine whether immediate SSC after CSs could also improve EBF by the end of six months after birth. Second, the newborns included in our study were healthy singleton and full-term deliveries. Further studies should be conducted to explore the impact of immediate SSC after CSs on higher risk mothers and newborns. Furthermore, in this current study, we were unable to control for some potential confounders that might have influenced breastfeeding outcomes, such as maternal breastfeeding intention before birth, lactation difficulties (including very small nipple, pulmonary adaptation syndrome in the newborn, insufficient production of breast milk, etc.), temperature at admission, etc. Without control for these confounders, it is possible that mothers who intended to breastfeed deliberately made an effort to spend more time on SSC after CSs and it was the intent to breastfeed that was responsible for the apparent advantage of SSC. Future studies examining these factors in details should be conducted.

## 5. Conclusions

Immediate SSC after CS was strongly associated with higher rates of EIBF. The EIBF rates increased with longer duration of SSC, with the at least 90 min SSC group having the highest EIBF rate (74.5%). Skin-to-skin contact is important for EIBF in Chinese baby-friendly hospitals. After multiple-testing correction, the rates of EBF at hospital discharge were found to be independent of the duration of SSC. SSC is suggested to be a clinically effective way to promote breastfeeding after CS. Our findings can be used to inform healthcare policy makers and providers for evidence-based decision making regarding SSC after CSs and are essential for designing targeted and effective breastfeeding promotion programs. More attention should be paid to ensuring immediate SSC is practiced after CSs to improve early breastfeeding initiation, and it is encouraged to provide SSC with longer durations to obtain the full benefit; if it is not feasible, a minimum of 30 min SSC could be performed to improve EIBF and EBF at discharge.

## Figures and Tables

**Table 1:** Maternal and neonatal characteristics of participants according to SSC duration after CS.

	No SSC (n = 136)	SSC Duration					
		<30 min (n = 84)	30–59 min (n = 168)	60–89 min (n = 146)	≥90 min (n = 145)	F/χ2	*p*
**Maternal Characteristics**							
Age (mean ± SD, years)	32.06 ± 4.66	32.06 ± 4.89	32.73 ± 4.46	32.21 ± 4.12	32.43 ± 4.19	0.59	0.67
Delivery gestational week (median (IQR), weeks)	39.14 (38.71–39.43)	39.29 (38.86–39.57)	39.14 (38.71–39.43)	39.00 (38.71–39.46)	39.00 (38.71–39.43)		0.14
Gravidity (median (IQR), times)	2 (1–3)	2 (1–4)	2 (2–3)	2 (1–3)	2 (2–3)		0.33
Parity (median (IQR), times)	1 (0–1)	1 (0–1)	1 (0–1)	1 (0–1)	1 (0–1)		0.92
Education level (n (%))						5.80	0.67
High school and less	28 (20.9%)	19 (22.9%)	27 (16.6%)	21 (14.4%)	25 (17.4%)		
College	84 (62.7%)	52 (62.7%)	112 (68.7%)	96 (65.8%)	99 (68.8%)		
Graduate and above	22 (16.4%)	15 (14.5%)	24 (14.7%)	29 (19.9%)	20 (13.9%)		
Height (mean ± SD, cm)	161.70 ± 4.81	161.44 ± 5.23	161.13 ± 4.90	160.66 ± 5.11	160.77 ± 4.90	1.05	0.38
Weight before delivery (mean ± SD, kg)	72.87 ± 10.18	72.94 ± 10.43	71.52 ± 8.66	69.81 ± 8.78	71.22 ± 8.95	2.47	0.05
**Neonatal Characteristics**							
Birth weight (mean ± SD, g)	3358.75 ± 396.15	3515.00 ± 443.41	3409.04 ± 383.94	3443.17 ± 444.90	3403.99 ± 385.05	2.11	0.08
Gender (n (%))						2.53	0.64
Female	67 (49.6%)	39 (46.4%)	77 (45.8%)	68 (46.6%)	78 (53.8%)		
Male	68 (50.4%)	45 (53.6%)	91 (54.2%)	78 (53.4%)	67 (46.2%)		
Birth length (mean ± SD, cm)	49.57 ± 1.43	49.73 ± 1.39	49.48 ± 1.39	49.64 ± 1.58	49.58 ± 1.34	0.51	0.73
Admitted to NICU or neonatal ward (n (%))	2 (1.5%)	0 (0)	1 (0.6%)	1 (0.7%)	2 (1.4%)	2.54	0.64

**Table 2:** Breastfeeding outcomes according to different SSC durations after CSs.

	No SSC	SSC Duration						
		<30 min	30–59 min	60–89 min	≥90 min	F/χ2	*p*	*P_adj_*
Early initiation of breastfeeding, n (%)	37 (27.2%)	25 (29.8%)	116 (69.0%)	108 (74.0%)	108 (74.5%)	120.34	<0.001	<0.001
Time to exhibition of feeding cues (median (IQR), min)	34.1 (21.4, 51.4)	18.2 (10.6, 35.9)	16.0 (10.0, 24.7)	14.0 (7.8, 26.8)	19.1 (9.9, 38.4)		<0.001	<0.001
Time to initiation of breastfeeding (median (IQR), min)	74.0 (60.0, 91.0)	68.5 (55.3, 89.0)	40.5 (24.0, 64.0)	38.5 (21.0, 62.3)	48.0 (30.0, 61.5)		<0.001	<0.001
Exclusive breastfeeding at hospital discharge, n (%)	70 (51.5%)	52 (61.9%)	111 (66.1%)	94 (64.4%)	100 (69.0%)	10.80	0.03	0.12
Mixed feeding at hospital discharge, n (%)	65 (47.8%)	30 (35.7%)	57 (33.9%)	51 (34.9%)	44 (30.3%)			
Formula feeding at hospital discharge, n (%)	1 (0.7%)	2 (2.4%)	0 (0)	1 (0.7%)	1 (0.7%)			
The age of the infant at discharge (median (IQR), day)	4 (3, 5)	4 (3, 5)	4 (4, 5)	5(4, 5)	4 (4, 5)	12.64	0.013	0.052

**Table 3:** Promoting factors for exclusive breastfeeding at hospital discharge.

	Model 1 *		Model 2 †	
	OR (95%CI)	*p*	OR (95%CI)	*p*
Maternal age (years)	1.00 (0.97, 1.04)	0.93	1.00 (0.96, 1.04)	0.89
Multiparity	1.63 (1.19, 2.25)	0.002	1.34 (0.94, 1.89)	0.10
Maternal weight before delivery (kg)	0.96 (0.94, 0.97)	<0.001	0.96 (0.94, 0.97)	<0.001
Macrosomia	0.38 (0.23, 0.64)	<0.001	0.53 (0.31, 0.92)	0.03
SSC duration				
No SSC at birth	1(Ref.)		1(Ref.)	
SSC duration <30 min	1.53 (0.88, 2.67)	0.13	1.62 (0.91, 2.86)	0.10
SSC duration 30–59 min	1.84 (1.16, 2.92)	0.01	1.86 (1.15, 3.01)	0.01
SSC duration 60–89 min	1.70 (1.06, 2.75)	0.03	1.79 (1.09, 2.91)	0.02
SSC duration ≥90 min	2.10 (1.29, 3.41)	0.003	2.16 (1.31, 3.56)	0.002
Time to exhibition of feeding cues (min)	1.00 (0.99, 1.01)	0.70	1.00 (0.99, 1.04)	0.55
Time to initiation of breastfeeding (min)	1.00 (0.96, 1.02)	0.38	1.00 (0.95, 1.02)	0.40

* Model 1 without any adjustment; † model 2 adjusted for maternal age, education, delivery gestational week and multiparity.

## Data Availability

The data presented in this study are available on request from the corresponding author.

## References

[1] Victora C.G., Bahl R., Barros A.J., França G.V., Horton S., Krasevec J., Murch S., Sankar M.J., Walker N., Rollins N.C. (2016). Lancet Breastfeeding Series Group. Breastfeeding in the 21st century: Epidemiology, mechanisms, and lifelong effect. Lancet.

[2] WHO Global Strategy for Infant and Young Child Feeding. http://apps.who.int/iris/bitstream/10665/42590/1/9241562218.pdf?ua=1&ua=1.

[3] UNICEF, WHO (2018). Capture the Moment: Early Initiation of Breastfeeding: The Best Start for Every Newborn.

[4] Khan J., Vesel L., Bahl R., Martines J.C. (2015). Timing of breastfeeding initiation and exclusivity of breastfeeding during the first month of life: Effects on neonatal mortality and morbidity--a systematic review and meta-analysis. Matern. Child Health, J..

[5] NEOVITA Study Group (2016). Timing of initiation, patterns of breastfeeding, and infant survival: Prospective analysis of pooled data from three randomised trials. Lancet Glob. Health.

[6] Nurokhmah S., Rahmawaty S., Puspitasari D.I. (2022). Determinants of Optimal Breastfeeding Practices in Indonesia: Findings From the 2017 Indonesia Demographic Health Survey. J. Prev. Med. Public Health.

[7] UNICEF (2021). The State of The World’s Children 2021 on My Mind, Promoting, Protecting and Caring for Children’s Mental Health.

[8] WHO Global Nutrition Targets 2025: Breastfeeding Policy. https://www.who.int/publications/i/item/WHO-NMH-NHD-14.7.

[9] Moore E.R., Bergman N., Anderson G.C., Medley N. (2016). Early skin-to-skin contact for mothers and their healthy newborn infants. Cochrane Database Syst. Rev..

[10] Safari K., Saeed A.A., Hasan S.S., Moghaddam-Banaem L. (2018). The effect of mother and newborn early skin-to-skin contact on initiation of breastfeeding, newborn temperature and duration of third stage of labor. Int. Breastfeed J..

[11] Lau Y., Tha P.H., Ho-Lim S.S.T., Wong L.Y., Lim P.I., Citra Nurfarah B.Z.M., Shorey S. (2018). An analysis of the effects of intrapartum factors, neonatal characteristics, and skin-to-skin contact on early breastfeeding initiation. Matern. Child Nutr..

[12] Cooijmans K.H.M., Beijers R., Brett B.E., de Weerth C. (2022). Daily skin-to-skin contact in full-term infants and breastfeeding: Secondary outcomes from a randomized controlled trial. Matern. Child Nutr..

[13] Li H.T., Luo S., Trasande L., Hellerstein S., Kang C., Li J.X., Zhang Y., Liu J.M., Blustein J. (2017). Geographic Variations and Temporal Trends in Cesarean Delivery Rates in China, 2008–2014. JAMA.

[14] Li L., Wan W., Zhu C. (2021). Breastfeeding after a cesarean section: A literature review. Midwifery.

[15] Hobbs A.J., Mannion C.A., McDonald S.W., Brockway M., Tough S.C. (2016). The impact of caesarean section on breastfeeding initiation, duration and difficulties in the first four months postpartum. BMC Pregnancy Childbirth.

[16] Prior E., Santhakumaran S., Gale C., Philipps L.H., Modi N., Hyde M.J. (2012). Breastfeeding after cesarean delivery: A systematic review and meta-analysis of world literature. Am. J. Clin. Nutr..

[17] WHO, UNICEF Implementation Guidance: Protecting, Promoting and Supporting Breastfeeding in Facilities Providing Maternity and Newborn Services: The Revised Baby Friendly Hospital Initiative, 2018. https://www.who.int/publications/i/item/9789241513807.

[18] WHO Global Breastfeeding Scorecard, 2017: Tracking Progress for Breastfeeding Policies and Programmes. 2017. https://www.who.int/publications/m/item/global-breastfeeding-scorecard-2017-tracking-progress-for-breastfeeding-policies-and-programmes.

[19] Qu W., Yue Q., Wang Y., Yang J.L., Jin X., Huang X., Tian X., Martin K., Narayan A., Xu T. (2020). Assessing the changes in childbirth care practices and neonatal outcomes in Western China: Pre-comparison and post-comparison study on early essential newborn care interventions. BMJ Open.

[20] Stevens J., Schmied V., Burns E., Dahlen H. (2014). Immediate or early skin-to-skin contact after a Caesarean section: A review of the literature. Matern. Child Nutr..

[21] Rowe-Murray H.J., Fisher J.R. (2002). Baby friendly hospital practices: Cesarean section is a persistent barrier to early initiation of breastfeeding. Birth.

[22] Karim F., Khan A.N.S., Tasnim F., Chowdhury M.A.K., Billah S.M., Karim T., Arifeen S.E., Garnett S.P. (2019). Prevalence and determinants of initiation of breastfeeding within one hour of birth: An analysis of the Bangladesh Demographic and Health Survey, 2014. PLoS ONE.

[23] Singh K., Khan S.M., Carvajal-Aguirre L., Brodish P., Amouzou A., Moran A. (2017). The importance of skin-to-skin contact for early initiation of breastfeeding in Nigeria and Bangladesh. J. Glob. Health.

[24] Li Z., Mannava P., Murray J.C.S., Sobel H.L., Jatobatu A., Calibo A., Tsevelmaa B., Saysanasongkham B., Ogaoga D., Waramin E.J. (2020). Western Pacific Region Early Essential Newborn Care Working Group. Association between early essential newborn care and breastfeeding outcomes in eight countries in Asia and the Pacific: A cross-sectional observational-study. BMJ Glob. Health.

[25] Widström A.M., Lilja G., Aaltomaa-Michalias P., Dahllöf A., Lintula M., Nissen E. (2011). Newborn behaviour to locate the breast when skin-to-skin: A possible method for enabling early self-regulation. Acta Paediatr..

[26] Agudelo S.I., Molina C.F., Gamboa O.A., Acuña E. (2021). Comparison of the Effects of Different Skin-to-Skin Contact Onset Times on Breastfeeding Behavior. Breastfeed Med..

[27] Zwedberg S., Blomquist J., Sigerstad E. (2015). Midwives’ experiences with mother-infant skin-to-skin contact after a caesarean section: ‘fighting an uphill battle’. Midwifery.

[28] Karimi F.Z., Sadeghi R., Maleki-Saghooni N., Khadivzadeh T. (2019). The effect of mother-infant skin to skin contact on success and duration of first breastfeeding: A systematic review and meta-analysis. Taiwan J. Obstet. Gynecol..

[29] Dominguez-Bello M.G., Costello E.K., Contreras M., Magris M., Hidalgo G., Fierer N., Knight R. (2010). Delivery mode shapes the acquisition and structure of the initial microbiota across multiple body habitats in newborns. Proc. Natl. Acad. Sci. USA.

[30] Bedford R., Piccinini-Vallis H., Woolcott C. (2022). The relationship between skin-to-skin contact and rates of exclusive breastfeeding at four months among a group of mothers in Nova Scotia: A retrospective cohort study. Can. J. Public Health.

